# Prediction of endotracheal tube size in pediatric patients: Development and validation of machine learning models

**DOI:** 10.3389/fped.2022.970646

**Published:** 2022-10-20

**Authors:** Miao Zhou, Wen.Y. Xu, Sheng Xu, Qing L. Zang, Qi Li, Li Tan, Yong C. Hu, Ning Ma, Jian H. Xia, Kun Liu, Min Ye, Fei Y. Pu, Liang Chen, Li J. Song, Yang Liu, Lai Jiang, Lin Gu, Zui Zou

**Affiliations:** ^1^ Department of Anesthesiology and Surgical Intensive Care Unit, Xinhua Hospital, Shanghai Jiaotong University School of Medicine, Shanghai, China; ^2^ School of Anesthesiology, Naval Medical University, Shanghai, China; ^3^ Department of Anesthesiology, Second Affiliated Hospital of Naval Medical University, Shanghai, China; ^4^ National Key Laboratory of Medical Immunology and Institute of Immunology, Naval Medical University, Shanghai, China; ^5^ School of Health Science and Engineering, University of Shanghai for Science and Technology, Shanghai, China; ^6^ Hebei North University, Zhangjiakou, China; ^7^ Department of Clinical Laboratory, 905th Hospital of PLA, Shanghai, China; ^8^ Department of Anesthesiology, Shanghai Pudong New Area People’s Hospital, Shanghai, China; ^9^ Department of Anesthesiology, Children's Hospital of Fudan University, Shanghai, China; ^10^ Department of Anesthesiology, First Hospital of Nanping City Affiliated to Fujian Medical University, Nanping, China; ^11^ Research Center for Advanced Science and Technology, The University of Tokyo, Tokyo, Japan

**Keywords:** endotracheal intubation, pediatric patients, endotracheal tube size, machine learning, prediction.

## Abstract

**Objective:**

We aimed to construct and validate machine learning models for endotracheal tube (ETT) size prediction in pediatric patients.

**Methods:**

Data of 990 pediatric patients underwent endotracheal intubation were retrospectively collected between November 2019 and October 2021, and separated into cuffed and uncuffed endotracheal tube subgroups. Six machine learning algorithms, including support vector regression (SVR), logistic regression (LR), random forest (RF), gradient boosting tree (GBR), decision tree (DTR) and extreme gradient boosting tree (XGBR), were selected to construct and validate models using ten-fold cross validation in training set. The optimal models were selected, and the performance were compared with traditional predictive formulas and clinicians. Furthermore, additional data of 71 pediatric patients were collected to perform external validation.

**Results:**

The optimal 7 uncuffed and 5 cuffed variables were screened out by feature selecting. The RF models had the best performance with minimizing prediction error for both uncuffed ETT size (MAE = 0.275 mm and RMSE = 0.349 mm) and cuffed ETT size (MAE = 0.243 mm and RMSE = 0.310 mm). The RF models were also superior in predicting power than formulas in both uncuffed and cuffed ETT size prediction. In addition, the RF models performed slightly better than senior clinicians, while they significantly outperformed junior clinicians. Based on SVR models, we proposed 3 novel linear formulas for uncuffed and cuffed ETT size respectively.

**Conclusion:**

We have developed machine learning models with excellent performance in predicting optimal ETT size in both cuffed and uncuffed endotracheal intubation in pediatric patients, which provides powerful decision support for clinicians to select proper ETT size. Novel formulas proposed based on machine learning models also have relatively better predictive performance. These models and formulas can serve as important clinical references for clinicians, especially for performers with rare experience or in remote areas.

## Introduction

Endotracheal intubation, a fundamental skill required for the practice of medicine, has been widely used in pediatric patients from emergency department to operating room (1, 2). Children have specific airway morphology and anatomy, and the physiological and airway responses of them are more complex and changeable (3, 4). Thus, selection of ETT size may be straightforward for adults, but is more inaccurate in pediatric patients. An excessive ETT size may result in laryngeal injuries, such as tissue edema, local ischemia and even subglottic stenosis. Oppositely, an under-estimulated ETT size may lead to hypoventilation, poor end tidal gas monitoring and leakage of anesthetic gases (5, 6). Therefore, choice of optimal ETT is an important guarantee for safe airway management especially in pediatric patients.

Various methods have been proposed for prediction of appropriate ETT size. A variety of formulas based on parameters of growth and development of children were available. Age-based formulas were the most frequently used. However, they have been reported to have some imprecision, because children's physical development is a physiological process in constant and individualized change (7). Many recent studies also confirmed the advantages of using ultrasound to select appropriate ETT size (3, 4, 8). Nevertheless, pediatric patients in the awake state cannot cooperate with the ultrasound examination. In addition, ultrasound prediction would not be suitable during intubation in emergency situations. Consequently, it is necessary to find an accurate, simple and individualized method to predict the optimal ETT size.

Machine learning, an important part of Artificial intelligence (AI), use advanced mathematical approaches to integrate complex association of clinical data and develop highly predictive algorithms for individualize predictions in real-time (9, 10). It has been used in multiple aspects of safe airway management in pediatric patients, including diagnosis and assessment of difficult airway, monitoring of ventilator parameters and ventilator-associated event, and risk prediction of airway adverse events (11–13). However, to our knowledge, there are no relevant studies to predict ETT size of pediatric patients by machine learning models at present.

Here, we sought to develop and validate machine learning models for ETT size prediction. The present study had 3 main objectives: first, to explore the best predictive machine learning models of ETT size; second, to derive new predictive formulas based on machine learning models; and third, to validate the machine learning models by comparison with traditional formulas, clinicians, and external verification.

## Methods

### Patient population and database

This retrospective study was performed with obtaining approval from the local ethics committee (no. XHEC-QT-2021-067). Patient identity remained anonymous, and informed consent was exempted due to the retrospective nature of the data acquisition. We retrospectively collected electronic medical record data of patients who underwent tracheal intubation from 5 centers between November 2019 and October 2021. Inclusion criteria included pediatric patients (aged 0–14 years, American Society of Anesthesiologists (ASA) status of I - III) who had undergone general anesthesia (leak evaluation was performed immediately after intubation for selecing the optimal ETT size), and who had a preoperative chest radiograph. The exclusion criteria were as follows: spinal abnormalities, tracheal and laryngeal pathologies, pulmonary disease (airway hyper-reactivity or bronchial asthma previous), and history of tracheostomy.

To develop machine-learning models, patients from Shanghai Xinhua hospital and Shanghai Changzheng Hospital were pooled together as an internal cohort. Additionally, data of patients from Children's Hospital of Fudan University, Shanghai Pudong New Area People's Hospital and First Hospital of Nanping City Affiliated to Fujian Medical University were used for external validation. In order to study rigor and clinical authenticity, we divided the pediatric patients into two data sets (cuffed and uncuffed group) both in internal and external cohort, and performed all the analysis independently.

After anaesthesia induction, all patients’ tracheas were intubated by cuffed or uncuffed ETT. Leak evaluation was performed immediately after intubation. The optimal ETT size was defined as that size which allowed an air leak around the tube at an inspiratory airway pressure of 15–30 cmH_2_O. If an air leak occurred at airway pressure of less than 15 cmH_2_O or there was no air leak above an airway pressure of 30 cmH_2_O, the ETT was exchanged for a larger one or smaller one. This process was continued until an optimal size was achieved. Accordingly, the final size of ETT recorded was the optimal ETT size (14–16).

The anesthesia records of patients were retrospectively investigated. Data were collected and extracted by specialized anesthesiologists not involved in data analysis. Age, sex, height, weight, BMI, BMI class, ideal BMI and final size of ETT (internal diameter) were recorded. Tracheal data (tracheal diameter at C6, C7, T2 level respectively, and distance from C6 to tracheal carina) were obtained from chest radiographs which were limited to a standard posteroanterior projection in flat supine position. All chest radiographs were derived from the picture archiving and communication system of medical record system. Tracheal data were measured at the mid-body of C6, C7 or T2 with electric caliper by three anesthesiologists, and the mean values were recorded (17–19).

### Machine learning and data Pre-processing

**Figure 1:**
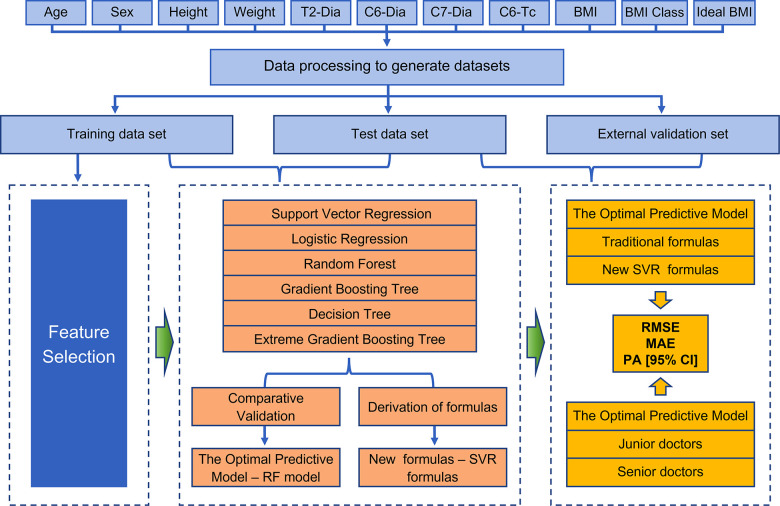
**Detailed flow chart of the entire study.** Patients with valid information were used as the total data set, and the data were processed to further derive training, test, and external validation sets. Feature selection was then performed, and six machine models were developed and validated to obtain the optimal predictive models, while new SVR formulas were derived to interpret the meaning of existing clinical formulas and they guide clinical decisions. Finally, the performance of the models was evaluated by assessing the metrics (RMSE, MAE and the prediction accuracy) when compared with both traditional formulas and clinicians.

Python (version 3.7.1.1) was used to build the prediction model. RF, GBR, DTR, SVR and LR analysis models were implemented using Python's scikit-learn package, while XGBR was implemented using Python xgboost package. The collected clinical cases were matched to generate cuffed and uncuffed datasets. Then the data were initialized and modifed to a uniform format. In order to get a higher quality data set, missing values were filled with the mean value based on age (20). The main prediction module used the Scikit-Learn machine learning library to train the model and predict the results. The data processing module used the Pandas machine learning library to pre-process the data set. The main process of machine learning can be illustrated in Figure 1.

### Feature selection

Ensemble models including extreme random tree (ET), gradient decision tree (GBDT), random forest (RF), and extreme gradient boosting tree (XGBR) were used to select the appropriate subset of features. First, continuous variables were transformed into four types of data: min-max normalization, z-score normalization, L2 normalization, and original data. Then, these four types of data were analyzed using the four algorithms mentioned above, and 16 models were constructed through 10 rounds of hierarchical cross-validation to obtain the median importance (final ranking of feature importance) of each variable in all models (14). Then, by using the RF algorithm, a new model is generated iteratively by adding one variable at a time, starting from the head of the variable ranking list (the most important variable), and calculating the classification accuracy. Finally, we selected the smallest list of features with the highest prediction accuracy.

### Evaluation metrics of models

All training sets were input into the model for retraining to obtain the final model, and the test sets were input into the model for testing. Mean absolute error (MAE) and root mean square error (RMSE) were utilized as evaluation metrics to assess the performance of machine learning algorithms as well as the prediction effectiveness. MAE is the mean absolute error of all samples. RMSE is the square root of the ratio of the square of the deviation of the predicted value from the true value to the number of observations. MAE and RESE value closer to 0 means the algorithm is better. Their statistics are defined as follows. In formulas, y_pre_ represents predicted value, y_true_ represents true value (optimal ETT size), and n represents the number of observations.

$$MAE=\frac{\sum |y_{pre}-y_{true}|}{n},\;RMSE=\sqrt{\frac{{\left(y_{pre}-y_{true}\right)}^{2}}{n}}$$

### Model development and generation

In this study, we used 6 models, including 2 linear (SVR and LR) and 4 non-linear machine learning models (RF, GBR, DTR and XGBR), to compare the performance of different models for ETT size prediction and ultimately developed the optimal performance prediction models for uncuffed and cuffed ETT respectively. Both uncuffed group data and cuffed group data were randomly divided into training and test sets in the ratio of 7: 3. The above models were trained in the training set using the training set feature values (minimum feature list). A Bayesian optimizer was used for internal validation. After training, predictions of models were tested in the test set. The prediction models with optimal performance were determined by comparing evaluation metrics. Subsequently, we further validated the optimal models in an external validation set. Equally important, the optimal linear models could be used to help us deduce novel formulas. It provided a reliable way for describing the growth law of children's tracheal diameter with mathematics.

### Comparison of optimal models and predictive formulas

The optimal models were selected to compare with traditional available formulas and our novel formulas for the predictive judgment of ETT size, and the evaluation metrics were MAE, RMSE and accuracy. In the comparison of uncuffed ETT size prediction, three traditional formulas were selected, they are presented as follows. First, Cole formula (21), ID (mm) = age/4 + 4. Second, Penlington formula (22): ID (mm) = age/4 + 4.5, when age is less than 6.5 years; ID (mm) = age/3 + 3.5, when age is greater than 6.5 years. Third, Height-based formula (23): ID (mm) = 2 + height/30, when age is from 3 months to 6 years. In the comparison of cuffed ETT size prediction, two traditional formulas were selected, they are presented as follows. Khine formula (24): ID (mm) = age/4 + 3, when age is less than 2 years; and Motoyama formula (25): ID (mm) = age/4 + 3.5, when age is 2 years or older. Age (in years) and height (in cm) for all formulas above.

### Comparison of optimal models and clinicians

Doctors including three junior and three senior clinicians, who participated in this study, predicted ETT size of patients in test set based on the variables collected retrospectively. They did not know the true ETT size throughout. And then the optimal models were compared with the predictive performance of clinicians in terms of predictive accuracy. Senior clinicians were defined as having more than or equal to 3 years’ experience of pediatric intubation, and senior clinicians were defined as having less than 3 years' experience in pediatric intubation.

### Statistical analysis

Continuous variables were presented as medians (interquartile range), and categorical variables were expressed as the number of cases or percentage. Python (version 3.7.1.1) was performed to conduct all machine learning models and to analyze data. Evaluation metrics of machine learning models and other methods were MAE, RMSE and prediction accuracy. Prediction accuracy was calculated with a 95% confidence interval (CI). Group differences of prediction accuracy were evaluated using chi-square tests, and *p* < 0.05 was used to indicate statistical significance.

## Results

### Study population

**Figure 2:**
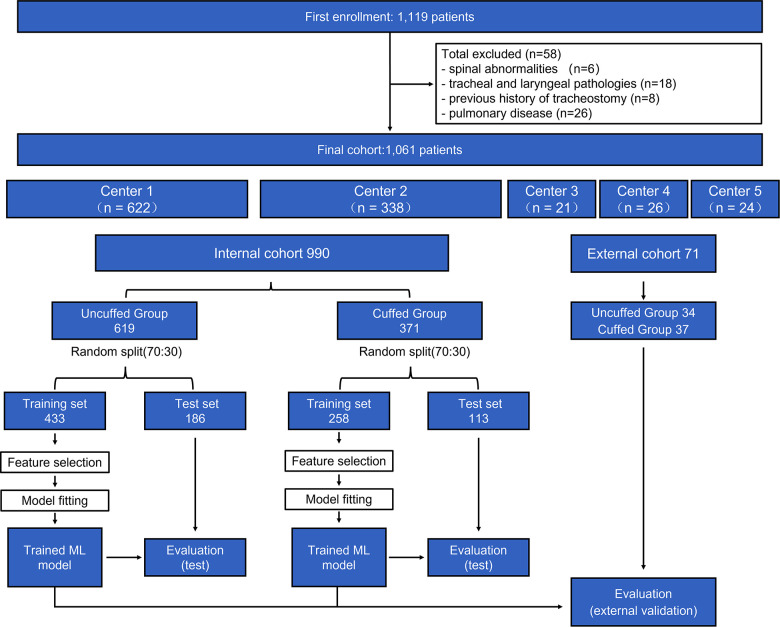
**Population flow chart.** In total, 1,061 patients were included in the final analysis (990 in internal cohort and 71 in external cohort). The cohort was further divided into uncuffed and cuffed groups, and each group was divided into training set and test sets. Machine learning (ML) models were built in the training set to perform feature selection and cross-validation. Developed models were then evaluated in the test set and external cohort. Center 1 = Shanghai Xinhua hospital; Center 2 = Shanghai Changzheng Hospital; Center 3 = Children's Hospital of Fudan University; Center 4 = Shanghai Pudong New Area People's Hospital; Center 5 = First Hospital of Nanping City Affiliated to Fujian Medical University.

The flow chart of the study is shown in Figure 2. During the study period, 1,119 pediatric patients were collected from the 5 centers. After excluding 58 patients, a total of 1,061 patients (990 in the internal cohort and 71 in the external cohort) were included in the final analysis. 619 and 34 patients were intubated with uncuffed ETT in the internal and external cohort respectively. The clinical characteristics of our study population are presented in Table 1. The median (interquartile range) age, weight and height of all patients included were 3.5 (1.5, 6.4) years, 15.0 (11.0, 23.0) kg and 100.0 (81.5, 120.0) cm respectively.

**Table 1:** Patient demographic data and variable features.

Variables	Internal Cohort (N = 990)				External Cohort (N = 71)	
	Uncuffed (N = 619)		Cuffed (N = 371)		Uncuffed (N = 34)	Cuffed (N = 37)
	Training set (*n* = 433)	Test set (*n* = 186)	Training set (*n* = 258)	Test set (*n* = 113)		
**Male**	280 (64.7)	121 (65.1)	145 (56.2)	76 (67.3)	23 (67.6)	17 (45.9)
**Age, years**	2.4 (1.1–3.9)	2.0 (1.0–4.0)	6.3 (4.2–8.9)	6.4 (4.3–9.3)	1.7 (0.7–3.2)	5.7 (3.1–8.1)
**Height, cm**	90.0 (76.5–104.0)	86.0 (73.0–105.0)	119.0 (105.0–133.0)	121.0 (102.0–135.0)	84.5 (71.3–97.8)	120.0 (98.0–130.0)
**Weight, kg**	12.5 (9.9–16.5)	12.5 (9.0–16.7)	22.9 (17.0–30.0)	23.0 (16.8–30.0)	11.2 (8.2–16.7)	21.0 (15.0)
**BMI, kg/m^2^**	15.9 (14.7–17.3)	16.2 (14.9–17.9)	16.2 (14.6–17.9)	16.2 (14.9–18.1)	15.6 (14.7–17.8)	16.6 (15.1–17.8)
**BMI Class**						
** I**	363 (83.8)	157 (84.4)	211 (81.8)	90 (79.6)	28 (82.7)	30 (81.1)
** II**	63 (14.6)	25 (13.4)	37 (14.3)	18 (15.9)	6 (17.6)	5 (13.5)
** III**	7 (1.6)	4 (2.2)	10 (3.9)	5 (4.4)	0	2 (5.4)
**Ideal BMI, kg/m^2^**	14.4 (12.9–16.2)	14.4 (12.9–16.2)	18.1 (15.8–20.0)	18.1 (15.8–20.0)	14.9 (6.7–16.4)	15.1 (8.0–17.3)
**ETT Size, mm**	5.0 (4.5–5.5)	5.0 (4.5–5.5)	5.5 (5.0–6.0)	5.5 (5.0–6.0)	5.0 (4.0–5.0)	5.0 (4.5–5.5)
**Tracheal diameter at T2 levels, cm**	0.8 (0.7–0.9)	0.8 (0.7–0.9)	1.0 (0.9–1.1)	1.0 (0.9–1.2)	0.7 (0.6–0.9)	1.0 (0.9–1.2)
**Tracheal diameter at C6 levels, cm**	0.7 (0.6–0.9)	0.7 (0.6–0.9)	0.9 (0.8–1.1)	0.9 (0.8–1.0)	0.7 (0.6–0.8)	0.9 (0.7–1.1)
**Tracheal diameter at C7 levels, cm**	0.8 (0.7–0.9)	0.8 (0.7–0.9)	0.9 (0.8–1.1)	0.9 (0.8–1.1)	0.7 (0.6–0.8)	0.9 (0.7–1.0)
**Distance from C6 to tracheal carina, cm**	5.9 (5.0–6.9)	5.9 (5.0–6.9)	7.8 (6.9–8.8)	7.8 (6.7–9.0)	5.6 (4.2–6.4)	7.7 (6.4–8.6)

Values are median (interquartile range) or *n* (%); BMI: body mass index.

### Feature selection

**Figure 3:**
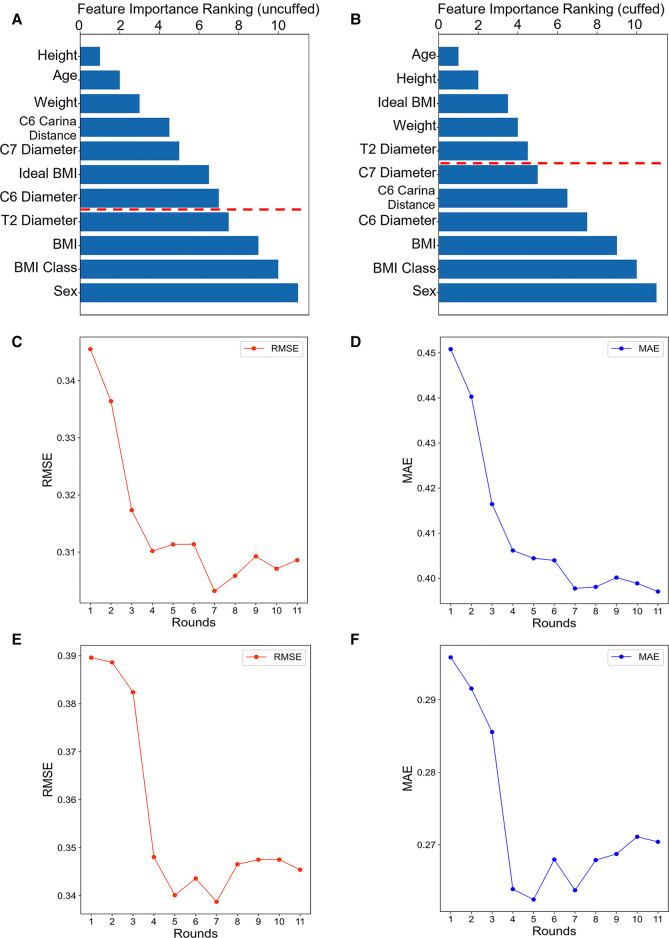
**Feature importance ranking and forward feature selection results.** The bar graph shows the importance ranking of variables usd for uncuffed ETT (**A**) and cuffed ETT (**B**) size prediction. The shorter the transverse column, the greater importance of the median ranking of the variable. The finally selected features were indicated above the red line. The line graphs show the forward feature selection results for uncuffed ETT (**C-D**) and cuffed ETT (**E-F**) size prediction. We examined the performance of the most prominent feature and identified the point at which there was no considerable decrease in RMSE and MAE, when adding the feature of the next highest ranking one to the model. As can be seen from the graph, the seventh feature is the lowest point in uncuffed ETT size prediction and the fifth is the lowest point in cuffed ETT size predciton. BMI: body mass index.

The importance ranking of all features were shown in Figures 3A,B. The minimum and optimal features were selected according to the RF model. We evaluated the predictive performance of the most prominent features and identified the cut-off at which there was no considerable decrease in RMSE and MAE when adding the feature of the next highest ranking one to the model. Finally, seven features (e.g., height, age, weight, tracheal length from C6 to carina, tracheal diameter at level of C7, ideal BMI and tracheal diameter at level of C6) were selected to be the optimal features in the uncuffed subgroup (Figures 3C,D). In the cuffed subgroup, five features (e.g., age, height, ideal BMI, weight, and tracheal diameter at level of T2) were screened out (Figures 3E,F). The significant features were listed above the red line in Figures 3A,B.

### Performance evaluation of different models

**Figure 4:**
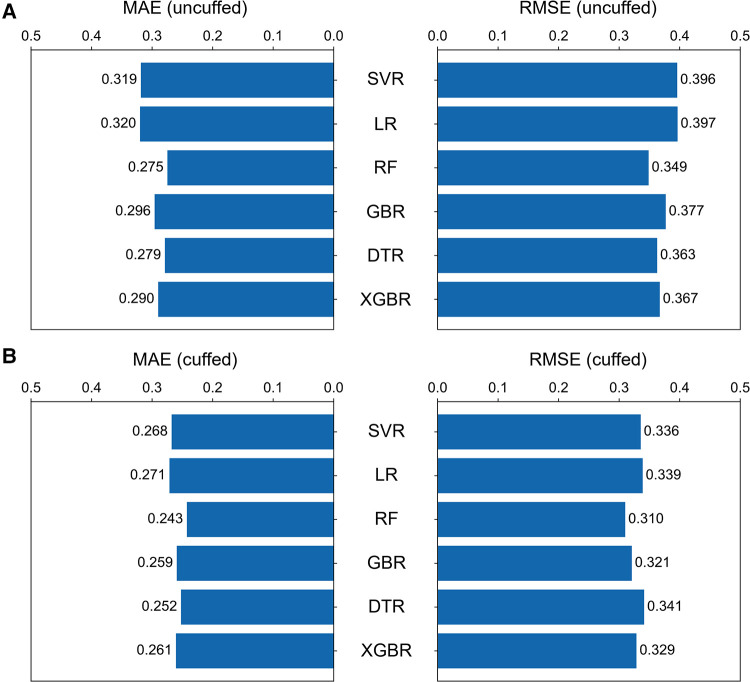
**Comparison of the six machine learning models.** The bar graphs showing the MAE and RMSE for uncuffed ETT (**A**) and the cuffed ETT (**B**) size prediction with six machine learning models. The random forest model performed the best of all models, and among the linear models, the support vector regression model performed better. SVR = support vector regression, LR = linear regression, RF = random forest, GBR = gradient boosting tree, DTR = Decision tree, XGBR = extreme gradient boosting tree.

Six machine learning models were developed based on the optimal features subset and their performances were compared. The RF models had the best performance in minimizing prediction error for prediction of both uncuffed and cuffed ETT size, and SVR was the better-performing linear models (Figure 4). In the test set, the performance of RF model for prediction of uncuffed ETT size was as follows: MAE = 0.275 mm and RMSE = 0.349 mm; and the prediction error of SVR model (MAE = 0.319 mm, RMSE = 0.396 mm) was lower than LR model (MAE = 0.320 mm, RMSE = 0.397 mm) (Figure 4A). Meanwhile, the cuffed ETT size RF predictor has a similar performance with MAE = 0.243 mm and RMSE = 0.310 mm; and the prediction error of SVR model (MAE = 0.268 mm, RMSE = 0.336 mm) was also lower than LR model (MAE = 0.271 mm, RMSE = 0.339 mm) (Figure 4B). Therefore, the RF models were selected as the final predictors to compare with traditional predictive formulas and clinicians. And SVR models were selected to derive linear predictive formulas.

### Derivation of formulas based on SVR machine learning model

According to feature selection and convenience of use, three formulas based on SVR models from complexity to simplicity were obtained in uncuffed and cuffed ETT size predictor respectively. Formula 1 has all the feature parameters. Formulas based on age, height, weight and formulas based on age only were proposed as formula 2 and formula 3. These formulas are presented as follows:

**SVR Formula 1 (uncuffed, 7 parameters):** ID(mm) = 2.34 − 0.0139 × age + 0.0264 × height − 0.00621 × weight − 0.0187 × C6 tracheal diameter + 0.577 × C7 tracheal diameter + 0.0388 × C6 to carina tracheal length − 0.0234 Ã— ideal BMI

**SVR Formula 2 (uncuffed, 3 parameters):** ID(mm) = 2.14 − 0.0314 × age + 0.0330 × height − 0.00752 × weight

**SVR Formula 3 (uncuffed, 1 parameter):** ID (mm) = 4.34 + 0.208 × age

**SVR Formula 1 (cuffed, 5 parameters):** ID(mm) = 3.34 + 0.131 × age + 0.00296 × height + 0.00682 × weight + 0.166 × T2 tracheal diameter + 0.0293 × ideal BMI

**SVR Formula 2 (cuffed, 3 parameters):** ID(mm) = 3.68 + 0.146 × age + 0.00491 × height + 0.00734 × weigh

**SVR Formula 3 (cuffed, 1 parameter):** ID(mm) = 4.09 + 0.200 × age

### Performance of optimal models and predictive formulas

**Figure 5:**
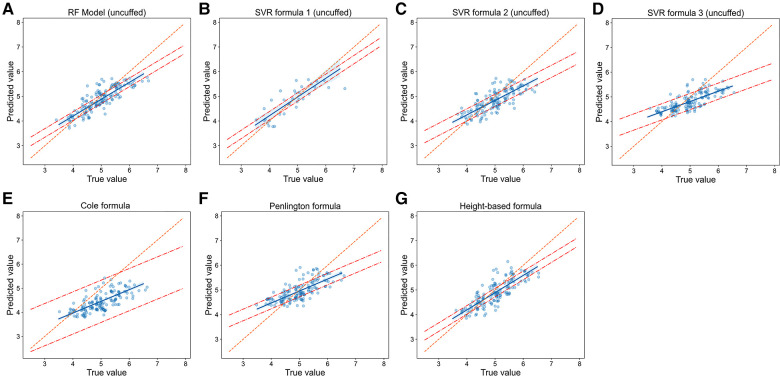
**Relationship of predicted values and true values in uncuffed ETT size prediction (model vs. Formulas).** Scatter plots show the relationship of optimal ETT size (x-axis) vs. predicted ETT size (y-axis) for uncuffed ETT size prediction with RF model (**A**), SVR formulas (**B-D**) and traditional formulas (**E-G**). The blue line represents the linear regression line, and the orange line represents the standard line for absolutely accurate prediction. The area between the two red dashed lines represents the 95% CI (confidence interval). RF = random forest, SVR = support vector regression.

**Figure 6:**
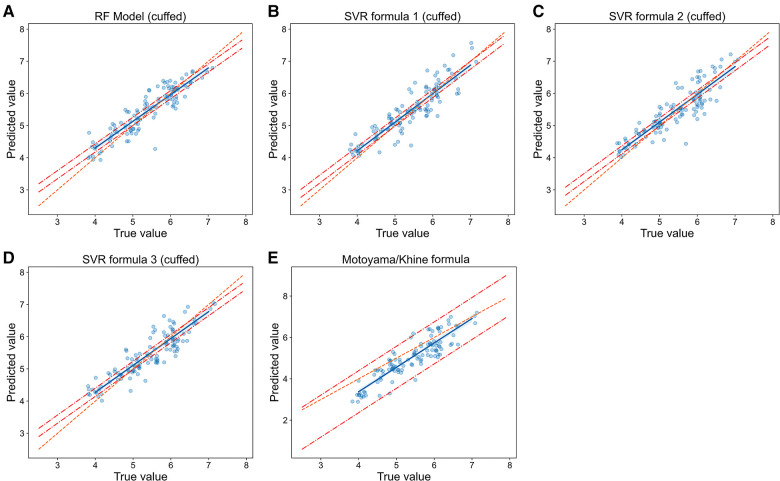
**Relationship of predicted values and true values in cuffed ETT size prediction (model vs. Formulas).** The scatter plots show the relationship of optimal ETT size (x-axis) vs. predicted ETT size (y-axis) for cuffed ETT size prediction with RF model (**A**), SVR formulas (B-D) and Motoyama/Khine formula (**E**). The blue line represents the linear regression line, and the orange line represents the standard line of absolutely accuracte prediction. The area between the two red dashed lines represents the 95% CI (confidence interval). RF = random forest, SVR = support vector regression.

We then compared the prediction error and accuracy of RF models and SVR formulas with traditional formulas (Table 2, Figure 5 and Figure 6). In terms of prediction of uncuffed ETT size, RF model had the best performance with the minimizing prediction error of MAE = 0.272 mm and RMSE = 0.343 mm and the highest accuracy of 52.3% (Table 2), and the regression line of machine learning model (Figure 5A) approached the line of identity more closely than formulas (Figures 5B–G); SVR formula 1 had a better performance with prediction accuracy = 50.3%; Cole formula had the worst prediction performance (MAE = 0.560 mm, RMSE = 0.666 mm and prediction accuracy = 17.4%). Comparison of RF model and formulas was performed in only 149 patients in test set, because height-based formula is applied with the age restriction (from 3 months to 6 years). We performed analysis on the whole patients (*n* = 187) in test set after height-based formula was removed, and the results showed that RF model also perform better than formulas (Supplementary Table 1). Thus, our RF model performed best in predicting uncuffed ETT size when compared with SVR formulas and traditional formulas.

**Table 2:** Validation of performance of the optimal machine learning models.

Precative methods	MAE	RMSE	Prediction accuracy [95% CI]
**Comparison of optimal models with predictive formulas**				
**Uncuffed (*n* = 149)**	**RF model**	0.272	0.343	52.3% [44.3–60.4]
	**Cole formula**	0.560	0.666	17.4% [11.4–23.5]^a^
	**Penlington formula**	0.305	0.395	45.6% [37.6–53.6]
	**Height-based formula**	0.297	0.364	46.3% [38.3–54.3]
	**SVR Formula 1**	0.317	0.400	50.3% [42.3–58.4]
	**SVR Formula 2**	0.314	0.388	45.6% [37.6–53.6]
	**SVR Formula 3**	0.356	0.443	38.3% [30.5–46.1]^a^
**Cuffed (*n* = 113)**	**RF model**	0.242	0.310	57.5% [48.4–66.6]
	**Motoyama/Khine formula**	0.473	0.572	29.2% [20.8–37.6]^b^
	**RF model**	0.242	0.310	57.5% [48.4–66.6]
	**SVR Formula 1**	0.268	0.336	54.9% [45.7–64.0]
	**SVR Formula 2**	0.267	0.331	52.2% [43.0–61.4]
	**SVR Formula 3**	0.263	0.328	53.1% [43.9–62.3]
**Comparison of optimal models with clinicians**				
**Uncuffed (*n* = 187)**	**RF model**	0.264	0.336	54.0% [46.0–62.3]
	**Junior doctors 1**	0.497	0.611	23.0% [16.1–29.8]^a^
	**Junior doctors 2**	0.375	0.489	34.8% [26.9–42.5]^a^
	**Junior doctors 3**	0.740	0.887	13.4% [7.7–18.7]^a^
	**Senior doctors 1**	0.306	0.460	49.2% [41.1–57.5]
	**Senior doctors 2**	0.295	0.435	49.2% [41.1–57.5]
	**Senior doctors 3**	0.340	0.500	45.5% [37.0–53.3]
**Cuffed (*n* = 113)**	**RF model**	0.251	0.308	57.5% [44.7–65.2]
	**Junior doctors 1**	0.489	0.613	27.4% [18.3–36.6]^b^
	**Junior doctors 2**	0.302	0.489	54.0% [43.6–64.1]^b^
	**Junior doctors 3**	0.401	0.562	37.2% [27.4–47.3]
	**Senior doctors 1**	0.269	0.423	54.9% [44.7–65.2]
	**Senior doctors 2**	0.275	0.419	53.1% [42.5–63.0]
	**Senior doctors 3**	0.236	0.403	61.1% [50.4–70.5]

RF, random forest; SVR, support vector regression; CI, confidence interval.

Data of 95% CI are presented as percentages.

^a^ Represents as *P* < 0.05 when compared with RF model in uncuffed ETT size prediction.

^b^ Represents as *P *< 0.05 when compared with RF model in cuffed ETT size prediction.

In cuffed ETT size prediction, RF model also performed best with MAE = 0.242 mm, RMSE = 0.310 mm and prediction accuracy = 57.5% (Table 2), and the regression line of machine learning model (Figure 6A) approached the line of identity more closely than formulas (Figures 6B–E); SVR formula 1 had a better performance with prediction accuracy = 54.9%, while MAE (0.268 mm) and RMSE (0.336 mm) were very close to SVR formula 2 (MAE = 0.267 mm, RMSE = 0.331 mm) and SVR formula 3 (MAE = 0.263 mm, RMSE = 0.328 mm); Motoyama/Khine formula had the worst prediction performance (MAE = 0.473 mm, RMSE = 0.572 mm and prediction accuracy = 29.2%). Thus, our RF model also performed best in predicting cuffed ETT size when compared with SVR formulas and traditional formulas.

### Performance of optimal models and clinicians

**Figure 7:**
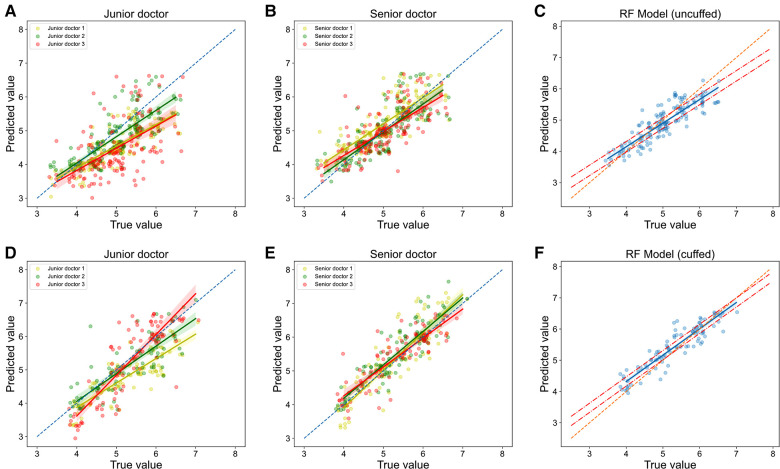
**Relationship of predicted values and true values in ETT size prediction (models vs. Clinicians)**. The scatter plots show the the relationship of optimal ETT size (x-axis) vs. predicted ETT size (y-axis) for uncuffed ETT (**A-C**) and cuffed ETT (**D-F**) size prediction with jonior clinicians (**A, D**), senior clinicians (**B, E**) and RF models (**C, F**). RF = random forest.

The performances of these models were then compared with clinicians. RF models performed slightly better than senior clinicians, while they significantly outperformed junior clinicians. In terms of prediction of uncuffed ETT size, MAE, RMSE, accuracy and accuracy within 5 mm of RF model prediction were 0.264 mm, 0.336 mm, 54.0% and 95.2% respectively, while total accuracy of senior clinicians and junior clinicians were 48.1% and 23.5%, and accuracy within 0.5 mm of them were 90.4% and 76.5% (Table 2 and Supplementary Table 2); The regression line of machine learning model (Figure 7C) approached the line of identity more closely than clinicians (Figures 7A-B).

In cuffed ETT size prediction, MAE, RMSE, accuracy and accuracy within 5 mm of RF model prediction were 0.251 mm, 0.308 mm, 57.5% and 99.1% respectively, while total accuracy of senior clinicians and junior clinicians were 55.8% and 39.8%, and accuracy within 5 mm of them were 92.0% and 83.2% (Table 2 and Supplementary Table 2); The regression line of machine learning model (Figure 7F) approached the line of identity more closely than clinicians (Figures 7D,E).

### External verification

Data of 71 patients were applied for external validation. Thirty-four of them were intubated with uncuffed ETT, and 37 with cuffed ETT. Comparison of prediction error and accuracy of RF models with formulas in external set is shown in Table 3. Similarly, RF models performed better in external verification. The performance of RF model in uncuffed ETT size prediction were MAE = 0.215 mm, RMSE = 0.284 mm and accuracy = 67.6%. In cuffed ETT size prediction, the performances of RF model were MAE = 0.327 mm, RMSE = 0.417 mm and accuracy = 45.9%.

**Table 3:** External validation of machine learning models.

		MAE	RMSE	Prediction accuracy [95% CI]
**Uncuffed (*n* = 34)**	**Cole formula**	0.423	0.510	35.3% [16.3–52.9]^a^
	**Penlington formula**	0.308	0.380	47.1% [27.0–65.3]
	**Height-based formula**	0.270	0.353	58.8% [38.7–76.7]
	**RF model**	0.215	0.284	67.6% [47.1–83.7]
**Cuffed (*n* = 37)**	**Motoyama/Khine formula**	0.331	0.409	40.5% [24.7–56.4]
	**RF model**	0.327	0.417	45.9% [29.9–62.0]

RF, random forest; CI, confidence interval.

Data of 95% CI are presented as percentages.

^a^ Represents as *P* < 0.05 when compared with RF model in uncuffed ETT size prediction.

## Discussion

Currently, there are several methods for prediction of ETT size, of which traditional predictive formulas are the most widely used in view of safety and convenience, but these formulas are not accurate and may produce conflicting results (8, 17, 26). In this multicenter retrospective study, we developed and validated machine learning models, which could be used to predict the optimal ETT size in pediatric patients. To our knowledge, the present study is the first to predict ETT size of pediatric patients using machine learning algorithms. There are three important findings in this study. First, the random forest models were identified to be the best models for predicting both uncuffed and cuffed ETT size. Second, based on machine learning models, we proposed three novel formulas for uncuffed and cuffed ETT size prediction, respectively. Third, the random forest models outperformed traditional formulas and clinicians in predicting ETT size.

In the present study, seven features (e.g., height, age, weight, tracheal length from C6 to carina, tracheal diameter at the level of C7, ideal BMI and tracheal diameter at the level of C6) were selected to be the optimal features subset in the uncuffed ETT size prediction, and five features (e.g., age, height, ideal BMI, weight, and tracheal diameter at the level of T2) were screened out to be the optimal features subset. In base of the results of feature selection, we proposed novel formulas using SVR models (the optima linear models) for uncuffed and cuffed ETT size prediction, respectively. Furthermore, formulas based on age, height and weight and formulas based on age only were proposed, due to easy availability of the three variables. The validation results showed that these formulas, with accuracy from 38.3 to 54.0%, performed relatively better than traditional formulas. Among them, multivariate-based formulas performed better than one-parameter formulas. The novel formulas we proposed could be used to account for the growth law of children's tracheal diameter in China and even Asia. Furthermore, these formulas provide important reference information to guide clinicians in prediction of ETT size.

We also compared RF models' performance (the optimal machine learning models) directly to the performance of traditional predictive formulas, and the results demonstrated that the RF models, with the accuracy of 52.3% to 57.5%, were much superior to the traditional formulas. This might be because traditional formulas are mostly linear formulas, while growth and development of children rise in a non-linear manner (27). It has previously been shown that the allometric growth curve can be used to account for the tracheal diameter in infants and young children. Apart from this, the other possible reason may lie in the fact that traditional formulas compared with our RF models are all one-parameter formulas, while RF models developed in base of multiple variables. Other scholars have also proposed multivariate-based formulas to improve predictive accuracy, but their clinical use has been limited since they involve more complex calculations (28). Our RF models can make accurate predictions in a matter of seconds, implying superior clinical utility.

However, we had expected our machine learning models and formulas to have higher accuracies. There are multiple possible reasons why the accuracies of our machine learning models and formulas were modest. First, patients with malnutrition or congenital disease (such as congenital heart disease and cleft lip/palate) show different growth patterns in airway anatomical structures (29–31). Our machine learning models might have performed better if the nutritional status and congenital disease of patients would have been considered in the development of machine learning models. Second, this was a retrospective study. We used a limited set of clinical variables when machine learning models were developed. And it is possible that increased accuracies can be achieved as more clinically relevant variables are added to our machine learning models.

Additionally, we compared our RF models to clinicians, since ETT size is selected eventually at the clinicians' discretion in clinical work. The results showed that RF models outperformed junior clinicians, while performed comparable to senior clinicians, and even slightly better than senior clinicians. In most hospitals, endotracheal intubation is commonly performed by senior clinicians, with limited opportunities for junior residents (32). And for some clinicians working in non-specialized hospitals, pediatric intubation is simply uncommon. The machine learning models we developed may provide a reliable basis and reference for junior clinicians or less experienced clinicians. Notably, the accuracy of senior clinicians' prediction in real clinical work may be higher than the result of the current study. The reasons may be as follows. Senior clinicians predicted ETT size based on the variables collected retrospectively in the present study, while they choose a suitable ETT size using other factors such as experience, other variables (e.g., the width of little finger), or even in intuition (33). This issue could be addressed in prospective future studies.

Selecting a suitable ETT size is a key step to ensure the success of pediatric intubation, and it is strictly related to clinical experience of intubation providers (34). Tracheal intubation is frequently performed in general anesthesia. In operating room, intubation is usually provided by senior anesthesiologist, while junior residents have less experience. Sometimes, in emergency department or intensive care medicine (ICU), emergency intubation may be required for critically ill pediatric patients, but those patients vary from only 0.1% to approximately 5% (35). Therefore, exposure to pediatric intubation is rare for clinicians in emergency department and ICU. In addition, the ability to intubate is also a basic skill required for clinicians in a setting of primary care center in the developing world or at remote locations, but they may have little experience of intubating pediatric patients. The infrequency of exposure creates substantial challenges, for clinicians, to develop a confident method to predict ETT size, and it may limit opportunities to minimize the risk of adverse events for patients (32). Prediction of ETT size by machine learning models in current study is non-invasive and quick. RF models can give predictive results in few seconds, with no need of specialist train or complex formulas. Our machine learning algorithms may play an important role in ETT size prediction for intubation providers, especially for non-specialist intubating pediatric ETT infrequently or in remote areas. Furthermore, RF models, as assistant tools, may be integrated into medical record system. In this setting, they can automatically provide predictive results of ETT size as references for clinicians, by identify patients' clinical data in medical record system.

There are some limitations in the present study to be considered. First, it was a retrospective study which might cause the loss of some clinical data. Despite Missing-data were substituted with mean values of missing items, the predictive performance of machine learning models may be improved by collecting more complete data in the future. In addition, retrospective data collection may be more prone to recording errors (e.g., ETT size and type of ETT). Thus, it seems reasonable to conduct prospective studies on this subject. Second, all the patients included in this study were of Chinese ethnicity, and therefore the generalization of the novel machine learning models and formulas to other ethnic groups is difficult. Third, we assumed that only one size of ETT was appropriate for each patient, but that was not the case. As pressure requiring to achieve air leakage around ETT differs in type of cuffed ETT (microcuff or others), future studies will continue to explore this issue. And whether RF model can be directly used for clinical decisions is yet to be confirmed in further prospective clinical studies.

In conclusion, our RF models demonstrated good performance for predicting optimal ETT size. They performed comparable to senior clinicians, while significantly outperformed traditional formulas and junior clinicians. Novel formulas proposed based on machine learning models also have relatively better predictive performance. These models and formulas can serve as important clinical references for clinicians, especially for performers with rare experience or in remote areas.

## Data Availability

The raw data supporting the conclusions of this article will be made available by the authors, without undue reservation.
