# Supplementary material for: Prediction of endotracheal tube size in pediatric patients: Development and validation of machine learning models
Source: Front Pediatr. 2022 Oct 20;10:970646. doi: 10.3389/fped.2022.970646 (PMC9631215; doi:10.3389/fped.2022.970646)
Supplement: Supplementary file 1 [file Table1.docx]

**Supplemental Table 1. Comparison of optimal model with formulas for uncuffed ETT size prediction in whole test set**

|  | | **MAE** | **RMSE** | **Prediction accuracy [95% CI]** |
| --- | --- | --- | --- | --- |
| **Uncuffed**  **(n=187)** | **Cole formula** | 0.517 | 0.634 | 21.9% [16.1-28.0] **^a^** |
|  | **Penlington formula** | 0.353 | 0.461 | 40.6% [33.8-47.9] **^a^** |
|  | **RF model** | 0.275 | 0.349 | 54.0% [46.6-60.9] |
|  | **SVR Formula 1** | 0.327 | 0.410 | 48.7% [41.2-55.6] |
|  | **SVR Formula 2** | 0.333 | 0.413 | 42.8% [35.9-50.1] **^a^** |
|  | **SVR Formula 3** | 0.382 | 0.473 | 35.8% [29.1-42.9] **^a^** |

RF, random forest; SVR, support vector regression; CI, confidence interval.

Data of 95% CI are presented as percentages.

a represents as *P* <0.05 when compared with RF model in uncuffed ETT size prediction.

**Supplemental Table 2. Comparison of optima models with clinicians in prediction accuracy**

|  |  | **Total correct rate** | **Rate of gap within**  **0.5 mm** | **Rate of gap larger**  **than 1.5 mm** |
| --- | --- | --- | --- | --- |
| **Uncuffed**  **(n=187)** | **Junior clinicians** | 23.5% **^a^** | 76.5% **^a^** | 6.4% **^a^** |
|  | **Senior clinicians** | 48.1% | 90.4% **^a^** | 0.5% |
|  | **RF model** | 54.0% | 95.2% | 0 |
| **Cuffed**  **(n=113)** | **Junior clinicians** | 39.8% **^b^** | 83.2% **^b^** | 1.8% |
|  | **Senior clinicians** | 55.8% | 92.0% **^b^** | 0.9% |
|  | **RF model** | 57.5% | 99.1% | 0.9% |

RF, random forest.

a represents as *P* <0.05 when compared with RF model in uncuffed ETT size prediction.

b represents as *P* <0.05 when compared with RF model in cuffed ETT size prediction.
